# Reducing Moisture Absorption in Polypropylene Nanocomposites for Automotive Headlamps Using Hydrophobicity-Modified Graphene/Montmorillonite

**DOI:** 10.3390/nano13091439

**Published:** 2023-04-22

**Authors:** Pyoung-Chan Lee, Dongwoo Kang, Ji Taek Oh, Jae Young Seo, Donghyeok Shin, Jae-Uk Jung, Youn Ki Ko, Jin Uk Ha, Myeong-Gi Kim

**Affiliations:** 1 Chassis & Materials Research Laboratory, Korea Automotive Technology Institute, Cheonan-si 31214, Republic of Korea; pclee@katech.re.kr (P.-C.L.);; 2 R&D Center, BESTGRAPHENE Co., Ltd., Yeoju-si 12616, Republic of Korea; 3 R&D Center, Woosung Chemical Co., Ltd., Cheonan-si 31214, Republic of Korea; 4 R&D Planning Department, Nifco Korea Co., Ltd., Asan-si 31409, Republic of Korea

**Keywords:** graphene, nanocomposites, montmorillonite, moisture

## Abstract

Polypropylene (PP) is used as a housing material in automotive headlamps but can cause fogging as a result of absorbed moisture and temperature differences between the exterior and interior of the housing. In this study, PP was combined with a graphene/montmorillonite hybrid (MMT-G) to yield a nanocomposite with reduced moisture absorption. Crucially, the modified nanofiller had low hydrophilicity and good compatibility with the PP matrix. Notably, the water contact angle of the MMT-G improved by 676%. Furthermore, the maximum moisture absorption of the PP/MMT-G nanocomposites was reduced by up to 11.22% compared to that of commercial PP composites, and the weight of the headlamp housing was decreased by 3.6%. Therefore, the designed nanocomposites are expected to help mitigate headlamp fogging while slightly reducing the housing weight.

## 1. Introduction

Automotive headlamps are key automotive components, particularly when forward visibility is poor. In the past decades, headlamps served as simple light sources; however, in recent years, their functions have diversified because of the increasing importance of vehicle appearance and higher safety demands. Accordingly, the internal geometry of the lamp is becoming more complex, and headlamp parts are increasingly prepared using polymers because of their high design freedom [1,2,3,4]. However, because of the added functions and more complex design, automotive headlamps have gradually become heavier. Therefore, strategies to decrease the weight and increase the rigidity of such parts should be developed. Further, the internal temperature increases and decreases when the lamp, which is enclosed, is turned on and off, respectively, which results in the release of the moisture contained in the polymeric housing and increases the humidity inside the lamp. Consequently, fogging occurs because of the temperature difference between the inside and outside of the housing, which causes performance deterioration and negatively affects appearance.

Several studies have analyzed the aforementioned fogging phenomenon. For example, Guzej and Zachar [2] used computational fluid dynamics (CFD) simulations with a simplified three-dimensional (3D) model of a headlamp to simulate phenomena related to fluid flow and phase changes. Watson et al. [1] described a technique for the transient numerical simulation of liquid film formation on surfaces during lighting, as well as thermal analysis, using CFD, including the effects of the properties of the liquid film on thermal solution. Singh et al. [3] implemented the Eulerian wall film (EWF) approach in a three-dimensional model to determine the underlying physics controlling the defogging phenomenon inside the headlamp. Furthermore, Drapala [4] proposed and evaluated several potential structural modifications to headlamps to reduce water condensation.

In addition, studies have been conducted to analyze the moisture absorption and desorption properties of polymeric materials that can cause fogging in automotive headlamps [5,6,7]. Bielecki et al. [5] proved that the condensation on the surfaces in the lamp did not solely originate from the water vapor present in the air, and the moisture in the plastic components of the lamp significantly contributed to this phenomenon. Notably, they found that the amount of moisture in the air increased upon the heating of the plastic lamp parts regardless of whether the lamp was ventilated. Therefore, condensation formation in automobile lamps seems to be directly linked to the hygroscopicity of the plastic housing. Later, Kim et al. studied the moisture absorption and moisture desorption of polymeric materials applied to exterior automotive lamps [6,7].

Polymers and plastics generally have good formability and can be used to prepare components with complex geometries through injection molding. In addition, polymeric materials are lighter than metals, which is advantageous for their application in automobiles. However, polymers are mechanically less resistant than metallic materials, which limits their applications. Moreover, most polymers absorb moisture, which changes their physical properties [6,8]. For products that are exposed to the atmosphere, investigations on their ability to absorb atmospheric moisture are significant. Crucially, the water absorbed by polymeric materials or composites causes structural changes as it penetrates the free volume of the polymer; for example, micropores are reversibly generated during moisture absorption and desorption [6,7].

Recently, international environmental and fuel-efficiency regulations have necessitated vehicle weight reduction; therefore, polymer composites are attracting increasing attention. Polypropylene (PP) is a general-purpose polymer that is used in various industries owing to its low cost and low specific gravity [9]. Currently, 70% of the thermoplastics used in vehicles are PP composites [9]. Among them, PP–talc composites are used in automotive parts such as headlamp housings because of their high formability and low cost. However, PP–talc composites, which are hydrophobic and have low moisture absorption per unit weight, are the heaviest plastic components of headlamps and are the main source of moisture. Furthermore, owing to the high density of PP–talc composites resulting from the use of heavy additives, weight reduction is required.

As explained above, the moisture that condenses inside headlamps originates from that contained inside the plastic components such as the housing or bezel [2,3,4]. Therefore, to mitigate condensation, the reduction of the moisture absorption of these components is necessary. This can be achieved by blending polymers with plate-like inorganic additives having aspect ratios sufficient to alter the diffusion path of water molecules [10,11,12,13].

In the past few decades, the application of polymer-based nanocomposite materials in various fields has been studied. Nanoscale reinforcing fillers include montmorillonite (MMT), mica, talc, carbon nanotubes, and graphene. Achieving a uniform dispersion of these materials in the polymer matrix is crucial to enhance the physical properties compared to those of microscale composite materials, and accordingly, research on nanofiller surface treatments is underway [10,11,12,14,15,16]. For example, Chen et al. [14] studied the mechanical, thermal, and tribological properties of polyamide (PA) nanocomposites prepared with amino-modified graphene oxide (GO). Further, Raji et al. [15] and Bee et al. [16] studied the use of silane-functionalized clay. In addition, studies on the moisture absorption properties of polymer/clay nanocomposites have been conducted [10,11,12]. However, previous studies have not considered the hydrophilic properties of the clays because of the high moisture-absorbing ability of the matrix in nanocomposites prepared using polar polymers. In contrast, for polymers with low moisture-absorbing ability, such as PP, the hydrophilic properties of the clay filler are expected to have a significant impact. In our previous study [17], we demonstrated the hygroscopic behavior of PP nanocomposites prepared with alkylated graphene. However, the mechanical properties of the chemically modified PP/alkylated graphene nanocomposites were poor and, therefore, require improvement.

In this study, flake-shaped nanofillers based on MMT and graphene were prepared for application in a PP nanocomposite with low moisture absorption and excellent mechanical properties. The moisture absorption characteristics, mechanical properties, and formability of the produced PP nanocomposite were investigated and compared with those of the current commercial PP–30 wt% talc composite.

## 2. Materials and Methods

### 2.1. Materials

Cloisite-Ca^2+^ was obtained from BYK-Chemie GmbH (Germany) and used as the MMT. Di-*n*-alkyl dimethyl ammonium chloride (DDAC, >95.0%, TCI, Tokyo, Japan) and *N*, *N*-dimethylformamide (DMF, >95.0%, TCI, Tokyo, Japan, 99.5%) were used without any pretreatment. For preparing PP, CB5108 grade (Korea Petrochemical Ind. Co., Seoul, Republic of Korea) and J370 grade (Lotte Chemical Co., Seoul, Republic of Korea) were mixed in a 50:50 weight ratio. For preparing the talc, we used the KCNAP400 grade material procured from KOCH (Seocheon-gun, Republic of Korea). For the production of chemically modified graphene (CMG), graphite flakes (150 μm grade) purchased from Graphene Supermarket (Ronkonkoma, NY, USA) were used.

### 2.2. Nanofiller Preparation

Before the preparation of a graphene/montmorillonite hybrid (MMT-G), MMT was first treated with organic agents. Specifically, organo-MMT was synthesized through a cation exchange reaction with DDAC. Briefly, 10 mL of HCl was added to 500 mL of deionized (DI) water and stirred well. Thereafter, 8 g of DDAC was dissolved in this solution, which was then stirred at 80 °C for 3 h. Meanwhile, 29.09 g of MMT-Ca^2+^ was added to 1300 mL of DI water and stirred vigorously at 80 °C for 1 h. Afterward, the resulting mixture was mixed with a pre-prepared DDAC solution and allowed to react vigorously at 80 °C for 12 h. The synthesized organo-MMT was centrifuged, washed several times with a mixed solvent of dimethylacetamide/DI water, washed again with DI water, and freeze-dried for 48 h to obtain organo-MMT.

Second, positively charged graphene (CMG^+^) capable of forming chemical interactions with the cationic nitrogen (N^+^) of organo-MMT was produced. For CMG^+^, GO was prepared using an improved method [18] reported previously [17,19]. Subsequently, a CMG^+^ colloid with high dispersibility was prepared with 4,4-oxidianiline. Thereafter, 1000 mL of a 0.1% GO aqueous solution was treated using an ultrasonic homogenizer for at least 2 h, and a solution containing 40 g of 4,4-oxidianiline in 1000 mL of DMF was prepared, mixed, and stirred at 90 °C for 40 h. After the reactant was cooled to room temperature, the precipitate was obtained using centrifugation, washed seven to eight times with acetone, filtered to obtain CMG^+^ (positively charged graphene), and then immediately suspended in ethanol. As a result, an electrostatic repulsive force was generated between the graphene monolayers owing to the positive charge (–N^+^) of the substituted 4,4-oxidianiline, and a highly dispersed functional graphene colloid was obtained.

Finally, organo-MMT was added to ethanol and stirred for 30 min with a homogenizer to prepare a 5 wt% dispersion. The CMG^+^ colloid was then further modified by the addition of different amounts of graphene (0.1 wt% (MMT-0.1G), 0.5 wt% (MMT-0.5G), 1.0 wt% (MMT-1.0G), and 3.0 wt% (MMT-3.0G)) and stirred with a homogenizer. During this process, adsorption via self-assembly occurred via electrostatic attraction. After the adsorption process, the coprecipitated materials were filtered under reduced pressure, separated, washed with ethanol, and dried for 24 h in an oven at 60 °C to obtain the MMT-G nano-hybrids.

### 2.3. Nanocomposite Preparation

Nanocomposites were fabricated using a extruder (twin screw type, 32 mm, *L*/*D* = 40/1, Unee Plus Co., Hwaseong, Republic of Korea). The temperatures of the twin screw extruder were set to 210 and 200 °C at the feeder and die hole, respectively, and the rotor speed was set to 400 revolutions per minute (rpm). First, polypropylene was supplied from the main feeder at 30 kg h^−1^, and the supply from the side feeder was such that the talc contents were 30 (reference sample) and 25 wt% (base sample). The PP/MMT-G nanocomposites were prepared by setting the base sample to be supplied at 30 kg h^−1^ from the main feeder, and that from the side feeder was supplied such that the MMT-G contents were 0.2, 0.3, 0.5, and 1.0 wt%.

Samples for the tensile tests (ASTM D638), flexural tests (ASTM D790), Izod impact strength tests (ASTM D256), and moisture absorption tests (80 mm × 80 mm, 1 mm (thickness)) were manufactured using an injection molding machine (NE80, Woojin Plaimm Co., Chungbuk, Republic of Korea).

### 2.4. Characterization and Instruments

The nanofillers were analyzed using scanning electron microscopy (SEM, Helios 5 Hydra CX, Thermo Fisher Scientific, MA, USA), Raman spectroscopy (Confotec MR350, SOL Instruments, Minsk, Belarus), zeta potential measurements (LITESIZER 500, Anton Paar, Graz, Austria), and Fourier transform infrared (FT-IR) spectroscopy (Spectrum Two, PerkinElmer, Waltham, MA, USA). To obtain the interlayer spacing of graphene and MMT-based fillers modified via different surface treatments, X-ray diffraction (XRD, small-angle X-ray scattering (SAXS) type, XEUSS 3.0 COMPACT, Xenocs, Grenoble, France) was performed on the MMT-G samples. The SAXS apparatus used Cu-*K*_α_ radiation (λ = 1.54 Å) generated at 50 kV and 0.6 mA. The data were collected between 2° and 10° in the 2*θ* mode. The water contact angle measurements were performed using a Phoenix-MT(T) system (Surface and Electro-Optics, Suwon, Republic of Korea) under ambient conditions. The mechanical properties (tensile properties (strength and elongation) and flexural properties (strength and modulus)), melt flow index, and Izod impact strength of the nanocomposites were measured using a universal testing machine (UT-100F, MTDI Korea Co., Daejeon, Republic of Korea), QM280 (Qmesys, Uiwang, Republic of Korea), and QM700L (Qmesys, Uiwang, Republic of Korea). The moisture absorption of the PP nanocomposites was calculated by measuring the weights before and after moisture absorption after leaving them in a constant temperature and humidity chamber (TH-G-180, Jeio Tech, Daejeon, Republic of Korea) at 80 °C and 85% relative humidity for 20,000 h.

Headlamp housings were also manufactured using the PP nanocomposites. The housing was shaped on a mold using a simplified form of the conventional mold and formed through an injection process. A 1050 t injection machine (LGH-1050M, LS Mtron, Anyang, Republic of Korea) was used, and the injection conditions were an injection temperature of 235–300 °C, mold temperature of 80 °C, injection pressure of 9 MPa, injection time of 4.3 s, holding pressure of 7 MPa, holding time of 8.0 s, and cooling time of 35.0 s. The molding characteristics were compared by analyzing the error relative to the design shape using a 3D measuring instrument (ATOS 2 Triple, GOM, Braunschweig, Germany).

## 3. Results and Discussion

### 3.1. Nanofiller Characterization

**Figure 1:**
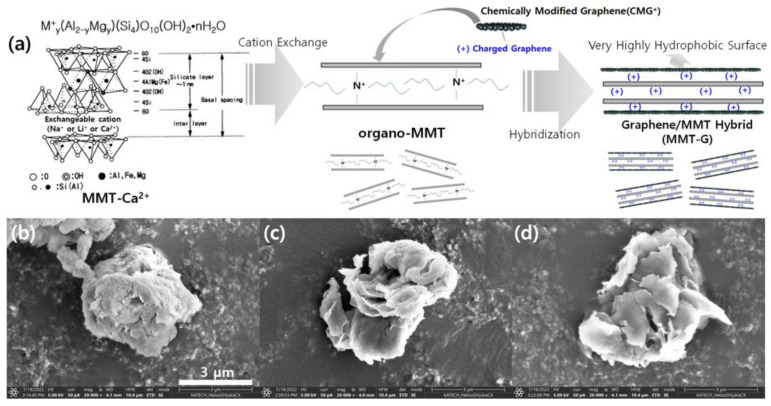
(**a**) Schematic of MMT-G fabrication and SEM images of (**b**) MMT-Ca^2+^, (**c**) organo-MMT, and (**d**) MMT-G.

MMT-Ca^2+^ is a type of smectite and silicate-layered clay mineral. In its structure, an alumina octahedral sheet is located between the silica tetrahedral sheets, and oxygen atoms are located between the silica tetrahedral sheets and alumina octahedral sheets [20,21]. MMT-Ca^2+^ achieves charge neutrality by intercalating Ca^2+^ cations between layers. Therefore, we designed MMT-G in which a graphene sheet was bonded to the silicate surface after the cations intercalated between the layers had been organically modified via ion exchange treatment. After the modification of these interlayers, the hydrophilicity of the interlayer structure is reduced, and the affinity for PP is increased. As a result, PP can be intercalated into the interlayer during compounding with the PP matrix. Moreover, the addition of hydrophilic MMT to a hydrophobic PP matrix is expected to increase the moisture absorption rate [12]. Therefore, as shown in Figure 1a, the organo-MMT surface was coated with positively charged graphene (CMG^+^) to reduce the hydrophilicity of the MMT and improve the mechanical properties. In addition, the graphene was bonded to the surface of the silicate, and the surface became hydrophobic, leading to a high affinity of both the interlayers and surfaces toward PP molecules.

Figure 1b–d show SEM images of various MMT samples. As shown in Figure 1b, MMT-Ca^2+^ is agglomerated without delamination. However, in the case of organo-MMT (Figure 1c), less aggregation is observed compared with MMT-Ca^2+^, which is expected to be intercalated or exfoliated when combined with PP. Furthermore, Figure 1d confirms that MMT maintains its shape without aggregation in the graphene hybrid. Based on a comparison with Figure 1c,d, the surface of organo-MMT is rough, whereas the surface of MMT-G is smooth because of the coating with graphene sheets.

**Figure 2:**
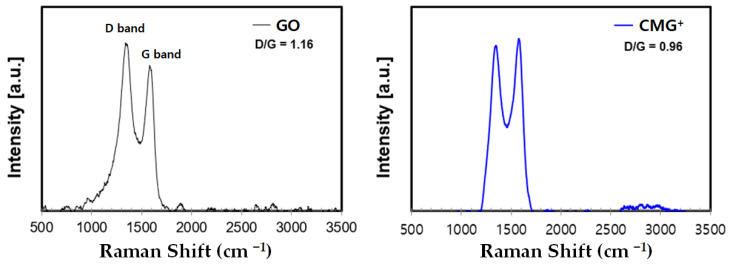
Raman spectra of GO and CMG^+^.

Figure 2 shows the Raman spectra of GO and CMG^+^, which are used to assess the structural features of the functionalized graphene used to fabricate MMT-G. Crucially, Raman spectroscopy is considerably useful for the analysis of graphene-containing materials. In particular, the disorder (D) peak (1360 cm^−1^) represents defects in the sp^2^ crystal structure of graphene, whereas the graphite (G) peak (1580 cm^−1^) represents the ordered (non-defective) C=C bonds and arises because of the vibrations of neighboring atoms in the hexagonal structure in the opposite direction [19]. Figure 2 shows the Raman spectra of GO and CMG^+^, and characteristic D and G bands are visible. Further, the intensity of the D band is high, probably as a result of functionalization. The D/G intensity ratio of GO is 1.16, whereas that of functionalized graphene (CMG^+^) is calculated to be 0.96, indicating that the structural defects are reduced via reduction and functionalization.

**Figure 3:**
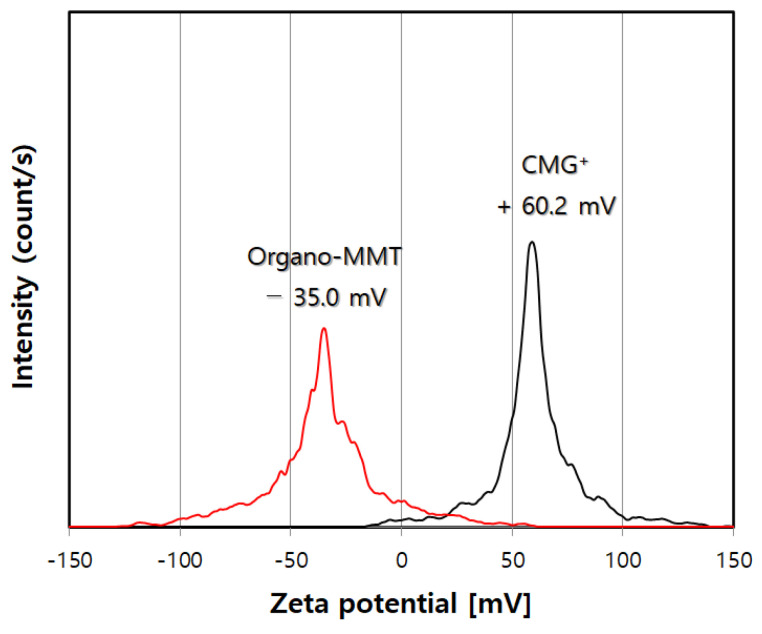
Zeta potentials of CMG^+^ and organo-MMT.

Figure 3 shows the zeta potential analysis results for CMG^+^ and organo-MMT. The sign and magnitude of the zeta potential provide information regarding the surface charge of a particle [19]. As shown in Figure 3, CMG^+^ has a strong surface positive charge (+60.2 mV) and, hence, adsorbs to organo-MMT (−35.0 mV), which has a negative charge on the silicate surface, via electrostatic self-assembly. In particular, zeta potentials higher than 30 mV are indicative of highly dispersible particles [19]. Therefore, based on its high zeta potential, CMG^+^ has a substantially high dispersion stability and a graphene structure comprising a monolayer and graphene with a few layers.

**Figure 4:**
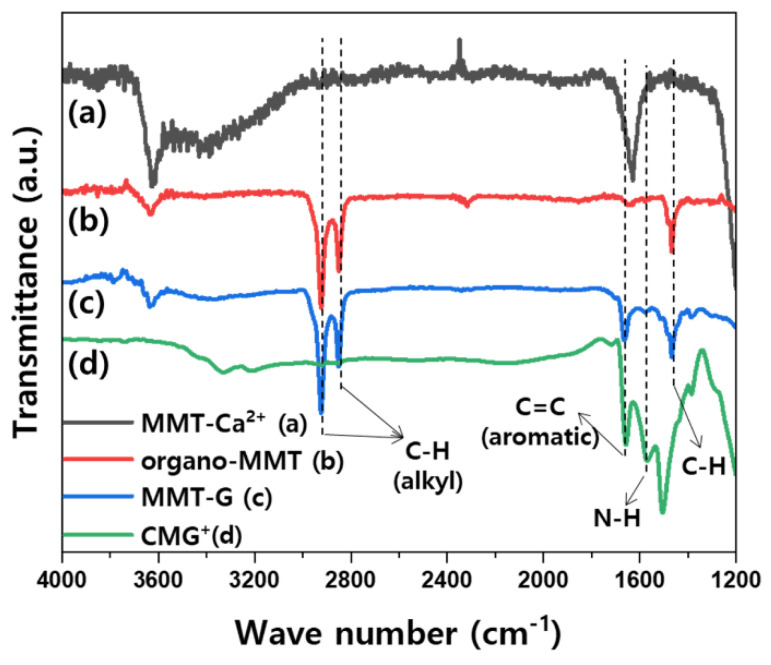
FT-IR spectra of MMT-Ca^2+^, organo-MMT, MMT-G, and CMG^+^.

Figure 4 shows the FT-IR spectra of the MMT-based nanofillers. As shown in Figure 4b, bands that can be assigned to the alkyl groups of DDAC appear at 2925 cm^−1^ and 2850 cm^−1^ as a result of the modification of MMT with this organic species. The FT-IR spectrum of MMT-1.0G (graphene content 1.0 wt%; Figure 4c) consists of bands at 1650 cm^−1^ (aromatic C=C) and 1570 cm^−1^ (N–H) arising from CMG (Figure 4d) [22,23,24]. In Figure 4a, bands corresponding to the stretching and bending of the –OH groups of adsorbed water are present at approximately 3430 cm^−1^ and 1630 cm^−1^, respectively. In contrast, these bands are significantly weak in the spectrum of organo-MMT (Figure 4b) because of the drying process used during MMT modification [25].

**Figure 5:**
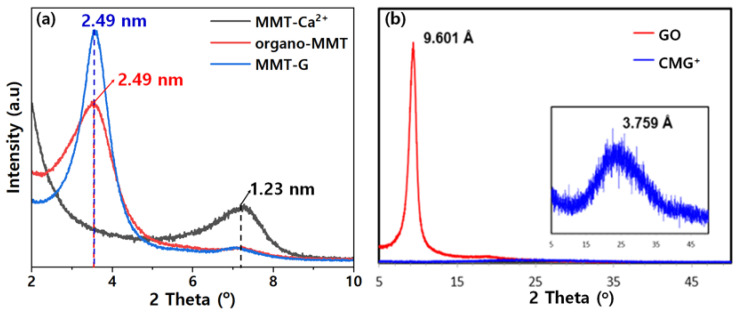
XRD patterns of (**a**) MMT-Ca^2+^, organo-MMT, and MMT-G and (**b**) GO and CMG^+^.

Figure 5 shows the XRD patterns of samples with different degrees of MMT functionalization used to investigate the interlayer structure of the MMT-based nanofiller. The distance between the layers of nanofiller was calculated using Bragg’s law [17,26]. As shown in Figure 5a, the *d*-spacing of organo-MMT was determined to be 2.49 nm, indicating an increase from that of MMT-Ca^2+^ (1.23 nm). Furthermore, the *d*-spacing of MMT-G was calculated to be 2.49 nm, which is similar to that of organo-MMT. Notably, the increase in the *d*-spacing of the nanofiller facilitated even dispersion in the PP matrix. The XRD patterns of GO and CMG^+^ are shown in Figure 5b, revealing graphene interlayer distances of 9.601 and 3.759 Å for GO and CMG^+^, respectively. Because graphene is obtained through the exfoliation of graphite, they have identical crystal structures, suggesting a layer spacing of 3.36 Å. Further, the layer spacing of GO increased owing to oxidation, whereas that of CMG^+^ narrowed again because of the small number of peripheral defects as a result of reduction and functionalization. In particular, the layer spacing of CMG^+^ was wider than that of graphite because of the introduction of functional groups, and the diffraction peak of CMG^+^ was also broad.

**Figure 6:**
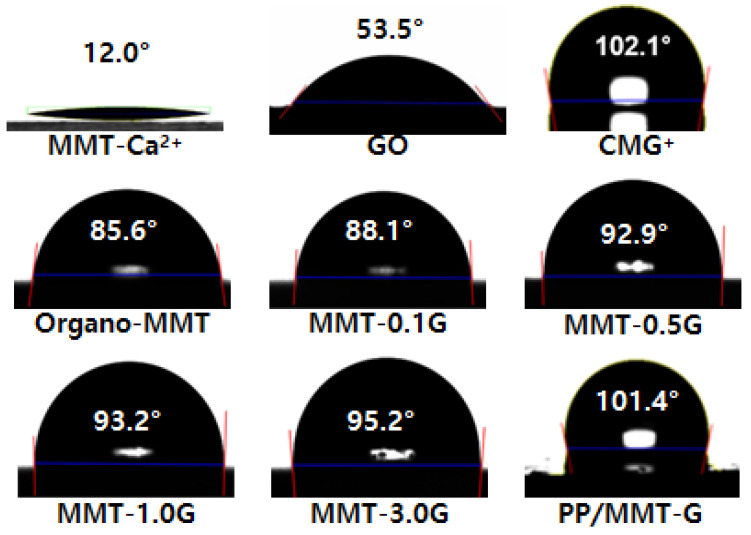
Contact angle measurements of MMT-Ca^2+^, GO, CMG^+^, organo-MMT, MMT-G (graphene contents of 0.1, 0.5, 1.0, and 3.0 wt%), and the PP/MMT-G nanocomposite.

Figure 6 shows images of the water contact angle measurements of MMT-Ca^2+^, GO, CMG^+^, organo-MMT, MMT-G nanofillers with various graphene contents, and the PP/MMT-G nanocomposite. The surface of MMT-Ca^2+^ is hydrophilic because of its structure (as discussed earlier), and as illustrated in Figure 6, owing to the hydrophilic properties of MMT-Ca^2+^, a substantially low contact angle (12.0°) was obtained. As previously reported by our research group [17], the surface of GO is hydrophilic because of the hydroxyl and carboxyl groups and exhibits a low water contact angle (53.5°). However, CMG^+^ is hydrophobic, which can be attributed to the reduction in the number of hydrophilic functional groups (hydroxyl and carboxyl groups) and the increase in the crystallinity of graphene.

In contrast to that of MMT-Ca^2+^, the water contact angle of the organo-MMT surface treated with DDAC was significantly improved. Moreover, as the amount of graphene in MMT increased, the water contact angle correspondingly increased, in addition to an increase in hydrophobicity. However, a higher graphene content results in a higher cost of the PP nanocomposite. Therefore, the determination of the optimal content is necessary. The 1.0 wt% graphene sample (PP/MMT-1.0G nanocomposite, contact angle of 101.4°) and 3.0 wt% graphene sample (PP/MMT-3.0G nanocomposite, contact angle of 101.7°) had similar contact angles. Therefore, in this study, we prepared PP nanocomposites with a graphene content of 1.0 wt% (MMT-1.0G).

### 3.2. Nanocomposite Characterization

**Figure 7:**
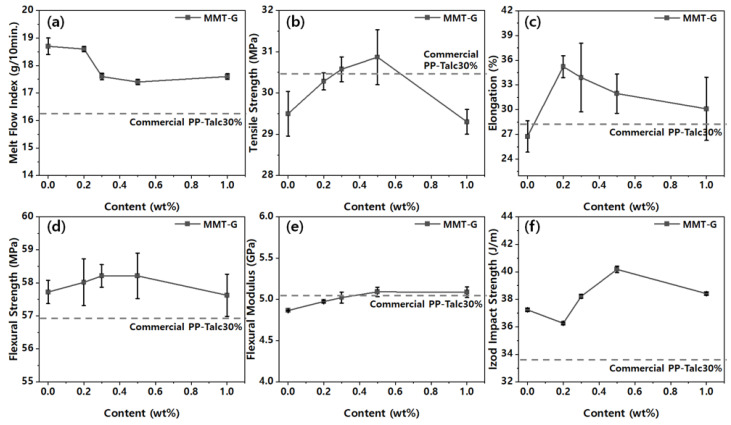
Mechanical properties of nanocomposites with respect to the nanofiller content: (**a**) melt folw index, (**b**) tensile strength, (**c**) elongation, (**d**) flexural strength, (**e**) flexural modulus, and (**f**) izod impact strength.

Figure 7 shows the mechanical properties of the PP nanocomposites in terms of the MMT-G content. As described earlier, MMT-1.0G (graphene content of 1.0 wt%) was used, and the standard used for mechanical property evaluation was commercial PP-30 wt% talc composite (data are shown by the dashed line in Figure 7).

As shown in Figure 7, the mechanical properties of the PP/MMT-G nanocomposites improved with the addition of the nanofillers, probably because of their surface-modifying effects that resulted in improved compatibility with the PP matrix via enhanced mechanical interlocking/adhesion at the nanofiller–matrix interface [26]. In general, the mechanical properties of polymeric nanocomposite materials depend on the size of particles, degree of dispersion, polymer-filler interfacial properties, and filler content [20]. The infiltration of the nanofiller also has a significant effect on the viscosity and melt flow of the composites and subsequent part processing [27]. As shown in Figure 7a, the melt flow index decreases with an increase in the nanofiller content, as frequently observed for nanocomposites. As expected, the tensile properties of the nanocomposites improved owing to the high rigidity of the nanofillers. Moreover, all physical properties of the PP/MMT-G nanocomposites were superior to those of the commercial PP–30 wt% talc composite. However, the specific gravity (1.10 g cm^−1^) of the PP/MMT-G nanocomposite (0.5 wt% in this study) was lower than that of the commercial PP–30 wt% talc composite (1.14 g cm^−1^). This should contribute to weight reduction when applied in vehicles.

**Figure 8:**
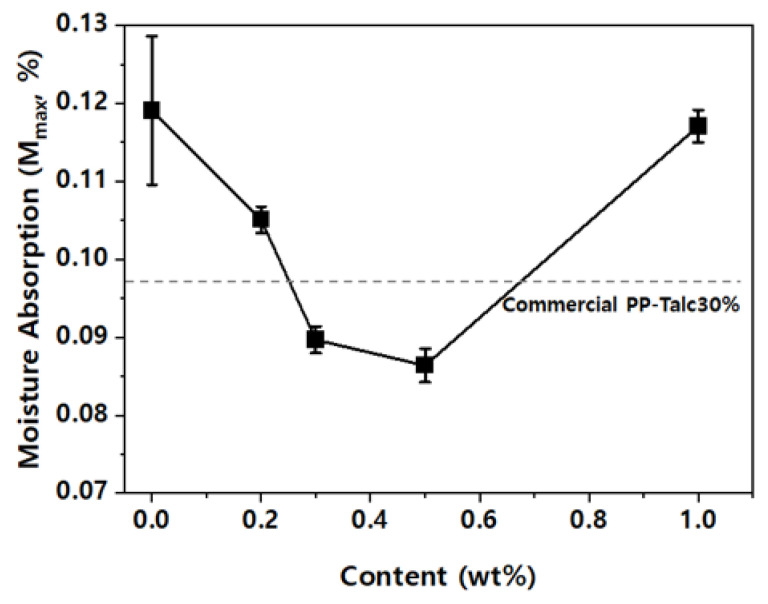
Maximum moisture absorption rate of nanocomposites with respect to the nanofiller content.

Figure 8 shows the *M*_max_ (maximum moisture absorption) of the PP nanocomposites with respect to the nanofiller content. Because of the hydrophobic properties of MMT-G and the interruption to the water diffusion path, the moisture absorption decreased with the increase in the MMT-G content. Specifically, for the PP/MMT-G nanocomposites, the moisture absorption decreased at low filler contents and then increased rapidly at significantly high filler contents (i.e., higher than 1.0 wt% MMT-G). This is because although the hydrophilicity of MMT is suppressed by the graphene coating, the hydrophilicity ratio (silicate ratio) increases above a certain level.

### 3.3. Application as a Headlamp Housing

**Figure 9:**
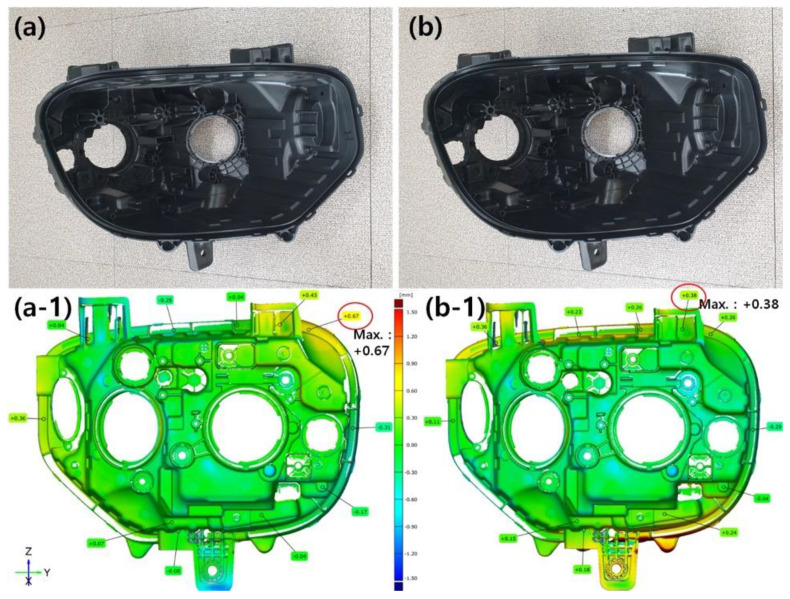
Digital photographs (**a**,**b**) and shape deviation color maps (**a-1**,**b-1**) used to evaluate the production accuracy of the headlamp housings prepared using (**a**,**a-1**) commercial PP composite and (**b**,**b-1**) PP/MMT-G nanocomposite.

Figure 9 shows images of headlamp housings prepared using the conventional PP composite and the nanocomposite prepared in this study. No significant differences were observed in processability or product shape between the materials. Further, because of the low specific gravity of the PP/MMT-G 0.5 wt% nanocomposite, the housing weight was 877.5 g, whereas the weight of the housing prepared using the commercial PP composite was 910.0 g. Therefore, the weight reduction rate was 3.6%.

In the preparation of headlamp housings, several factors influence the dimensional accuracy of the design [28]. Therefore, for forward engineering, in the initial design, dimensional tolerances are considered to reflect variations in the manufacturing process. In particular, polymer composites exhibit different degrees of shrinkage or twisting after injection depending on the shape or type of the additive. Therefore, the dimensional accuracy of the prepared parts was determined using coordinated measurements using a 3D measuring instrument after injection manufacturing. Specifically, a 3D image was obtained by measuring along a predetermined path to determine deviations in the dimensions of the produced parts from those of the computer-aided design (CAD) model. Figure 9a-1,b-1 show the shape deviation color map derived for the headlamp housings prepared using the conventional composite and nanocomposite, respectively. Notably, the measured maximum deviation is 0.38 mm for the commercial PP composite and 0.67 mm for the PP/MMT-G nanocomposites. Therefore, the dimensional accuracy of the PP/MMT-G nanocomposite is higher than that of the conventional PP composite.

## 4. Conclusions

In this study, graphene and MMT-based nanofillers were developed to reduce the moisture absorption of the PP composites used to prepare automotive headlamp housings. As a result of the hydrophilicity control facilitated by the use of the nanofiller, the moisture absorption of the PP nanocomposites was lowered by up to 11.22% (i.e., for the MMT-G 0.5 wt% nanocomposite). Overall, considering the moisture absorption, mechanical properties, and cost, the PP/MMT-G 0.5 wt% nanocomposite showed the optimal performance. In particular, the PP/MMT-G 1.0 wt% nanocomposite showed the lowest maximum moisture absorption among the analyzed samples. However, this composite is unsuitable for industrial applications because of its poor mechanical properties. Nevertheless, by manufacturing a headlamp housing with the derived composition, a weight reduction of 32.5 g (3.6%) compared to that of the product prepared using the commercial PP–30 wt% talc composite was achieved. Therefore, the designed nanocomposites are expected to help mitigate headlamp fogging while reducing weight.

## Data Availability

The data presented in this study are available on request from the corresponding author.
